# Giant Conjunctival Nevus in a 12-Year-Old Child

**DOI:** 10.1155/2017/8414352

**Published:** 2017-08-23

**Authors:** Edit Tóth-Molnár, Eszter Vizvári, Ákos Skribek, András Vörös

**Affiliations:** ^1^Department of Ophthalmology, University of Szeged, 10-11 Korányi Fasor, Szeged 6720, Hungary; ^2^Department of Pathology, University of Szeged, 2 Állomas Street, Szeged 6725, Hungary

## Abstract

We describe a case of a giant conjunctival nevus presented in a 12-year-old girl with suspicious clinicomorphological appearance. The lesion was noticed by the parents at the age of 3 years as a “fleshy spot” on the bulbar conjunctiva. The lesion remained unchanged until approx. 6 months before recent admission. On slit-lamp examination, a large conjunctival lesion with variegate pigmentation and indistinct margins was detected on the superonasal part of the bulbar conjunctiva of the left eye. Intralesional cysts and vessels were detected with AS-OCT examination. Wide excision and cryotherapy to the scleral bed were performed and amniotic membrane graft was used to restore the ocular surface. Histopathological examination revealed compound type conjunctival nevus and disclosed any sign of malignancy. Although giant conjunctival nevus is a rare entity, precise diagnosis and adequate management are very important as it can be confused with malignant melanoma.

## 1. Introduction

Conjunctival nevi are the most frequently diagnosed benign melanocytic tumors of the ocular surface. Considering the great variability of the clinical appearance, the diagnosis can be challenging. Large conjunctival nevi can cause further diagnostic and differential-diagnostic difficulties as they can be confused with malignant melanoma or—in amelanotic cases—with lymphangioma [1, 2]. Giant conjunctival nevus is a rare entity representing approximately 5% of conjunctival nevi according to the only available survey of 32 cases published by Shields and colleagues [3]. They used the terminology of “giant nevus” for the conjunctival nevi of 10 mm or greater in basal diameter. We report a rare case of a rapidly growing giant conjunctival nevus in a 12-year-old girl with suspicious clinicomorphological appearance.

## 2. Case Presentation

A 12-year-old girl was referred to our department with a pigmented conjunctival lesion suspicious for malignant melanoma by another ophthalmic hospital. The lesion was noticed by the parents when the child was 3 years old as a “fleshy spot” on the conjunctiva. The lesion remained unchanged until approximately 6 months before recent admission. During these months, considerable increase in the size of the alteration was noticed by the parents. Parts of the lesion became pigmented and elevated causing foreign body sensation.

**Figure 1:**
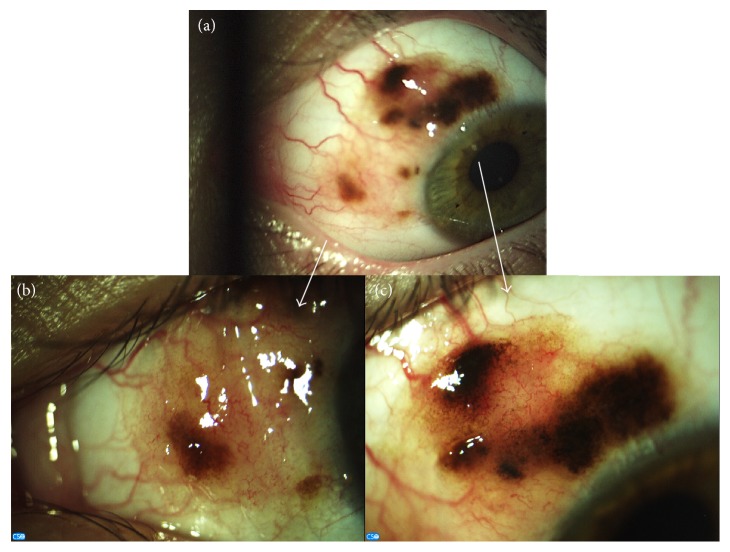
Clinicomorphological appearance of a giant conjunctival nevus (a). Nasal (b) and superonasal (c) parts of the lesion.

Detailed ophthalmological examination was performed. The visual acuity was 6/6 in both eyes without correction, and intraocular pressure was 12 Hgmm in the right and 13 Hgmm in the left eye. On slit-lamp examination, a large conjunctival lesion with variegate pigmentation and indistinct margins was detected on the superonasal part of the bulbar conjunctiva of the left eye (Figure 1). Enlarged feeding vessels were also observed. The anterior surface of the right eye and the ocular media and the fundi of both eyes were without pathological alterations.

**Figure 2:**
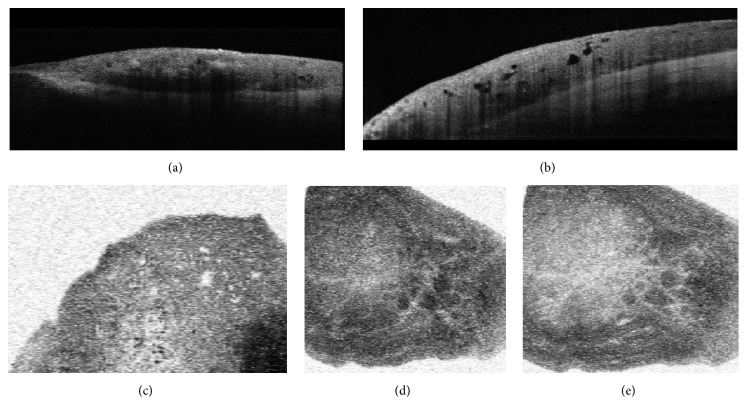
Results of anterior segment optical coherence tomography of conjunctival nevus. B scan images (a, b) show distinct posterior borders of various parts of the lesion with variegate degree of posterior shadowing and with numerous intralesional cystic lesion (b); C scans (c–e) can detect intralesional cysts (c) and intralesional vessels (d, e).

Anterior segment optical coherence tomography (AS-OCT) was performed with Topcon 3D 2000 OCT using three-dimensional (3D) volume scans. Variegate severity of posterior shadowing and multiple intralesional cysts were detected on B scans, whereas C scans revealed the presence of intralesional cysts and intratumoral vascularity (Figure 2).

**Figure 3:**
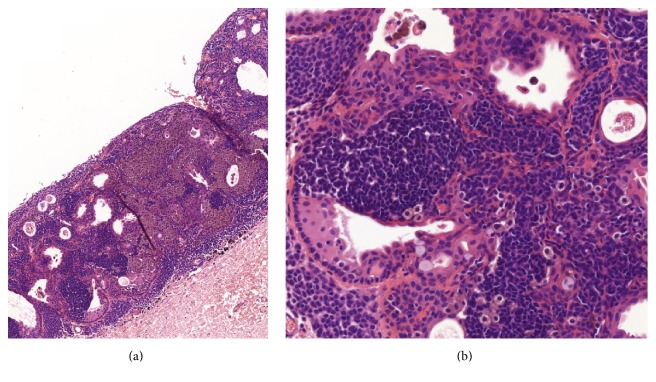
Histological examination revealed nevus cell nests with vertical maturation tendency and junctional activity in the epithelial-stromal border of the conjunctiva. Multiple cysts and intratumoral vessels could also be detected (magnification: (a) 10x, (b) 20x).

Written informed consent was obtained from the parents prior to surgery and also for the use of amniotic membrane. Excision of the tumor and cryotherapy for the scleral bed was performed under local anesthesia. Amniotic membrane graft was used for the reconstruction of the ocular surface. The resected tissue was admitted to histopathological examination. Histological examination revealed nevus cell nests with vertical maturation tendency and junctional activity in the epithelial-stromal border of the conjunctiva. Multiple cysts could also be detected. Neither pagetoid spread nor mitotic activity could be observed. The found histopathological signs were characteristic for compound type conjunctival nevus (Figure 3).

## 3. Discussion

Giant nevi represent a rare subgroup of melanocytic conjunctival alterations. Considering the extended conjunctival involvement and variegated clinicomorphological appearance, these lesions can cause differential-diagnostic difficulties as they can be confused with malignant melanoma. Although the potential of conjunctival nevi for malignant transformation is low (less than 1%), giant conjunctival nevi require careful management [4].

In our case, an enlarging conjunctival lesion with indistinct border and variegate pigmentation was detected on the bulbar conjunctiva in a 12-year-old girl. Rapid growth and profound change in pigmentation were noticed by the parents. These changes could be initiated by hormonal influences since melanocytic lesions of the conjunctiva tend to modify during puberty. The lesion raised the suspicion of malignant transformation, as (1) rapid enlargement was reported by the parents; (2) variegate pigmentation and indistinct margin of the lesion could be observed; (3) large feeding vessels were presented [5]. Although the clinicomorphological appearance raised the suspicion of malignancy, the patient's young age (conjunctival melanoma is extremely rare in the first two decades of life) and the intralesional cysts detected with AS-OCT B and C scan examinations predicted benign nature of the lesion. In accordance with the conclusion of the preoperative evaluations, histopathological examination revealed compound type conjunctival nevi and excluded malignancy. Epithelial cell-walled intralesional cysts are the “memory” of epithelial origin in conjunctival nevi; therefore they can be considered as signs of chronicity suggesting benign nature of the lesion [1, 2]. Intralesional cysts can be rarely seen in conjunctival melanoma although malignant melanoma arising from preexisting cystic nevi can feature intralesional cysts [6]. AS-OCT examination proved to be a very informative tool in the diagnosis of conjunctival nevi [7]. 3D volume scans can be analysed both cross-sectionally (B scans) and in the frontal plane, that is, perpendicular to the visual axis and so-called “en face” (C scans) [8]. B scan images can detect cysts in the cross-sectional scans, while intralesional vessel morphology can be exactly depicted on C scans allowing precise follow-up of intralesional vascularity. This latter finding can be of great importance as better visualization of intralesional morphology can lead to a better understanding of the natural course of nevus evolution. During follow-up examinations, alterations in the vascular network can be visualized and compared in various segmentation levels. Any sign of cystic or vascular alteration can be detected with great accuracy.

In conclusion, giant conjunctival nevi represent a rare subgroup of primary melanocytic lesions of the conjunctiva. AS-OCT can detect intralesional cysts and vessels within the nevi; therefore AS-OCT is a useful examination method in the management of conjunctival nevi. In suspicious cases, surgical excision must be performed to exclude malignancy.
